# An Overview of Rare and Unusual Clinical Features of Bietti’s Crystalline Dystrophy

**Published:** 2014

**Authors:** Ali Osman Saatci, Hasan Can Doruk

**Affiliations:** Department of Ophthalmology, Dokuz Eylul University, Izmir, Turkey

**Keywords:** Bietti’s crystalline dystrophy, cornea, macula, optical coherence tomography, retina

## Abstract

Bietti’s crystalline dystrophy (BCD) is a rare disease presenting with the appearance of intraretinal crystalline deposits and varying degrees of chorioretinal atrophy commencing at the posterior pole. Within time, intraretinal crystals gradually disappear and chorioretinal atrophy extends beyond the macula even resulting in complete chorioretinal atrophy. Concomitant corneal crystals can be noted in 1/2 - 1/3 of the patients, and the presence of corneal crystals is not a must for establishing the diagnosis. For the past decade, genetic evaluations and newer imaging modalities expand our knowledge about the disease. CYP4V2 gene is found to be the gene responsible for the disease process and new mutations are still being described. Modern imaging modalities, such as a spectral domain optical coherence tomography (SD-OCT) shed light on the anatomic features of the disease. By this, we reiterate the rare and unusual clinical features of BCD.

## INTRODUCTION

In 1937, Bietti (1) described three cases of glistening, yellow- white intraretinal crystals in the posterior pole, atrophy of the retinal pigment epithelium (RPE), choroid sclerosis, crystals in the superficial paralimbal cornea with onset in the third decade of life. The condition was named after him, and subsequently, more cases with “Bietti’s crystalline dystrophy” (BCD) have been reported in the majority of ethnic groups. Its prevalence is 3% for all retinitis pigmentosa (RP) patients and 10% for autosomal recessive RP patients (2). Bietti crystalline dystrophy is, by all odds, related to aberrant oxidation of cellular lipid metabolism (3), caused by mutations of p450 genes (4). The disease is thought to be inherited in an autosomal recessive manner, and familial cases have also been documented (5, 6). Genetic mutations of the CYP4V2 gene are detected in >93.4% of cases by sequence analysis (7) and thus far, more than 50 mutations have been described (8). Lai et al. (9) evaluated the genotype - phenotype analysis in BCD in a group of 18 Chinese patients in 13 families and showed that BCD with homozygous IVS6-8del17 bp/insGC or compound heterozygous IVS6-8del17 bp/insGC and IVS8-2A_G mutations appeared to have a more severe disease phenotype based on electrophysiological testing. In contrast, Rossi et al (10) described the clinical and genetic features of 15 Italian patients with BCD and illustrated that there was a large range of genotypic and phenotypic variations stressing out the lack of an explicit genotype-phenotype correlation. Wilson and colleagues (11) found crystals resembling cholesterol or cholesterol esters in the retina and complex lipid inclusions in the conjunctiva, cornea, fibroblasts and circulating lymphocytes. Lipid inclusions were also demonstrated in the choroid (12).

## CLINICAL FEATURES

Clinical findings are mainly characterized by fundus changes described below and the presence of crystalline corneal deposits.

*Fundus Findings*

Clinical findings are mainly characterized by fundus Stamp of the disease is the presence of refractive polygonal, yellowish-white intraretinal crystals grouped around the posterior pole and at the transition zone between the normal and atrophic RPE (13-15). During the disease, crystals diminish in number and atrophy of the RPE and choriocapillaris begin to initially appear at the central fundus. With the time, chorioretinal atrophy expands centrifugally spreading to the retinal periphery as well. For that reason, patients between the second and fourth decade of life experience vision impairment, some degree of night blindness and visual field defects. However, age of onset, presenting symptoms and disease severity vary widely from case to case, and there can also be asymmetry between the eyes. Deterioration of vision can become so severe that the patient may even end up in legal blindness by the fifth or sixth decade of life. Patients can be staged into three groups -- early, intermediate and advanced disease, according to the clinical evaluation as suggested by Yuzawa et al. (16) and used later by Mataftsi et al. (2) and Fang et al. (15) with some minor modifications.

**Figure 1:**
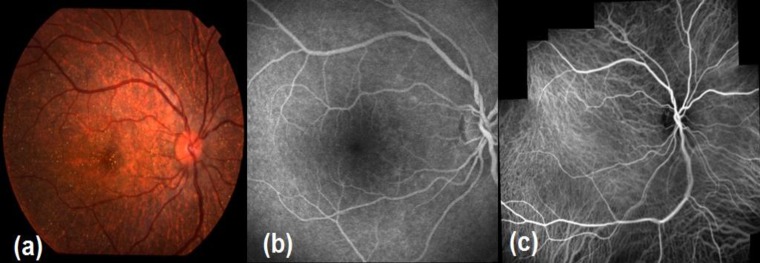
Early disease, color fundus (1a), fluorescein angiographic (1b) and indocyanine green angiographic appearance (1c)

*Early disease:* Crystals are scattered throughout the posterior pole and mid periphery. On fluorescein and indocyanine angiographies atrophy is limited to the posterior pole solely (Fig. 1a, b and c).

**Figure 2:**
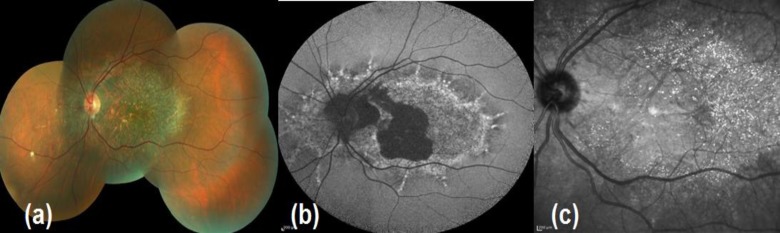
Intermediate disease, color fundus (2a), auto-fluorescence image (2b) and infrared image (2c)

*Intermediate disease:* Crystals are very few or absent at the posterior pole, but still visible outside the area of central atrophy extending up to the equator (Fig. 2a and b).

**Figure 3:**
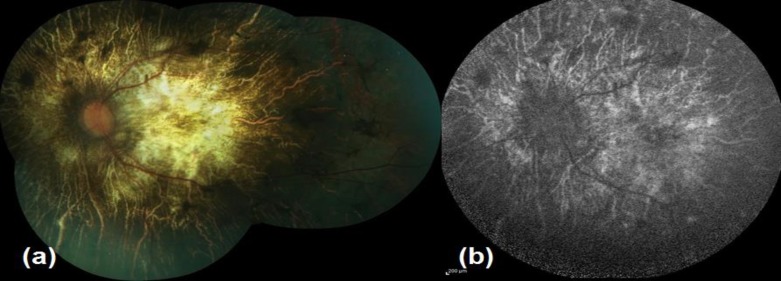
Advanced disease, color fundus (3a) and auto-fluorescence image (3b)

*Advanced disease:* There is almost complete chorioretinal atrophy with very occasional crystals (Fig. 3a and b).

**Figure 4:**
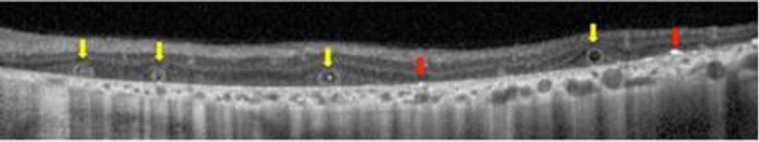
Outer retinal tubulations (yellow arrow), Bright plaque (red arrow)

**Figure 5:**
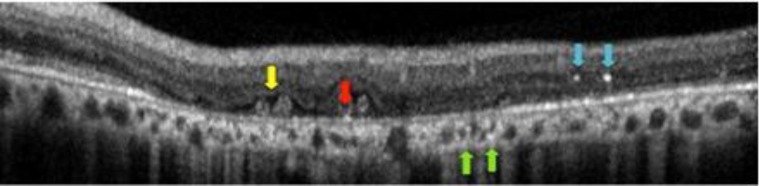
Outer retinal tubulations (yellow arrow), Bright plaque (red arrow), Intraretinal bright spots (blue arrow), Choroidal bright spots (green arrow)

**Figure 6:**
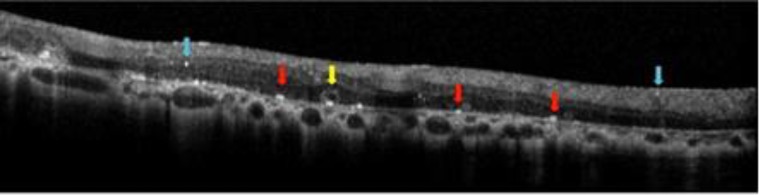
Outer retinal tubulations (yellow arrow), Bright plaque (red arrow), Intraretinal bright spots (blue arrow)

Besides the ancillary tests such as fluorescein and indocyanine angiographies, newer imaging modalities such as spectral domain optical coherence tomography (SD-OCT) and auto-fluorescence imaging reveals additional morphological features of the disease process such as outer retinal tubulations (ORT) and hyper-reflective dot-like lesions localized in almost all retinal layers (8, 17-22) (Figure 4-6). Outer retinal tubulations are more frequently described in BCD when compared with other retinal dystrophies such as RP and cone dystrophy (20, 21). 

Only in Yin’s et al. study comprising 17 patients, ORT was present in just 13.6% of eyes with BCD (23).Countless bright reflective spots of various configurations such as highly reflective spots in the inner retina, bright reflective plaques on top of Bruch membrane or partially encapsulated reflective plaques are all shown in SD-OCT sections of patients with BCD (19). However, only some of the hyper-reflective dots seem to correspond to the crystalline deposits (17, 19). The rest of the hyper-reflective dots may also be related to the inflammatory cells, protein deposits, a glial response to retinal degeneration, or even be artefacts (8).

Toto et al. (22) argued that choroidal hyper-reflective dots were in fact crystals resided in the choroid, thus they just added to the controversy of presence of hyper-reflective dots in the choroid.

Auto-fluorescence imaging shows decreased auto-fluorescence corresponding to the areas of RPE loss; punctual increase of auto-fluorescence corresponds to pigment dots, possible RPE hyperplasia or limited hyperauto-fluorescence of the crystals (17).

Trailing the evolving disease, macular function deteriorates as well. Some very rarely encountered macular changes may further affect the visual acuity devastatingly. Subfoveal neurosensory detachment (24), subretinal neovascular membrane (25-28), macular hole (29, 30) and cystoid macular edema (31, 32) are among those.

As the diagnosis mainly relies upon clinical features, electrophysiological tests are not as mandatory as in other retinal dystrophies. However, electrophysiological tests can be helpful to show the extent of the damage. The full field ERG can show extent of rod and cone dysfunction ranging from normal to reduced amplitudes of scotopic and photopic response to undetectable stimuli (33). On the other hand, multifocal ERG may detect regional areas of abnormal function wherever the full field ERG is normal (34).

Differential diagnosis of intraretinal crystalline deposits includes primary hyperoxaluria type 1 and 2, cystinosis, Sjögren-Larsson syndrome, drug-toxicity (tamoxifen, methoxyflurane and canthaxanthin) and talc retinopathy (7).

*CORNEAL FEATURES*

**Figure 7:**
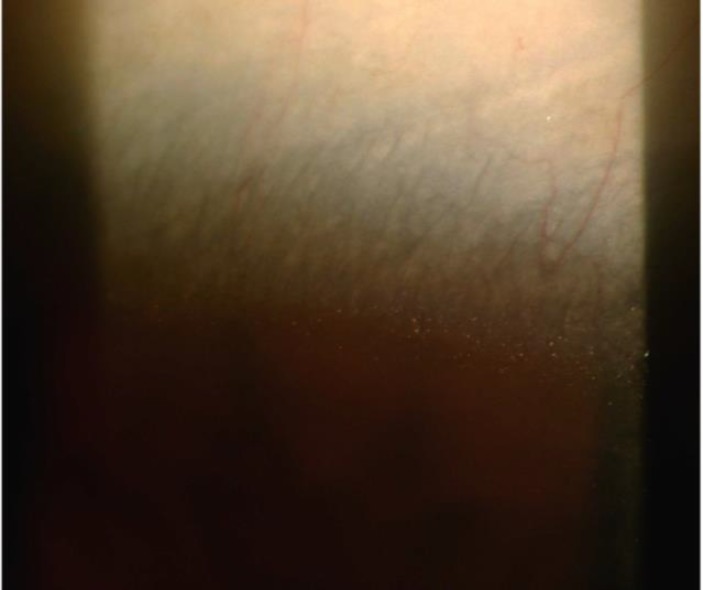
Limbal Crystals

Corneal crystals (figure 7) are very fine and located mainly in the subepithelial and superficial anterior stroma near the limbus. In 1/3 - 1/2 of cases can be observed at the slit-lamp examination (12). However, corneal crystals can easily be overlooked, even by an experienced ophthalmologists as these deposits are very subtle (35). Specular microscopy (36) and in vivo confocal microscopy (37) were used to detect the corneal crystalline deposits very recently.

## CONCLUSION

The diagnosis of BCD is made by the typical clinical findings of discrete glistening crystals within the retina. After excluding other causes of crystalline retinopathy with the help of clinical history and systemic evaluation diagnosis is confirmed by finding of the mutations in CYP4V2 gene. New imaging modalities such as SD-OCT provide novel information about the anatomic location and clinical implications of retinal crystals.
